# Characterizing Foxp3^+^ and Foxp3^-^ T cells in the homeostatic state and after allo-activation: resting CD4^+^Foxp3^+^ Tregs have molecular characteristics of activated T cells

**DOI:** 10.3389/fimmu.2024.1292158

**Published:** 2024-01-25

**Authors:** Zilei Liu, Katherine J. Baines, Natalie M. Niessen, Munish K. Heer, David Clark, G. Alexander Bishop, Paul R. Trevillian

**Affiliations:** ^1^ Transplant Unit, John Hunter Hospital, Newcastle, NSW, Australia; ^2^ Transplant and Surgical Immunology Theme, Immune Health Research Program, Hunter Medical Research Institute, The University of Newcastle, Newcastle, NSW, Australia; ^3^ School of Medicine and Public Health, College of Medicine, Health and Wellbeing, The University of Newcastle, Newcastle, NSW, Australia; ^4^ School of Biomedical Sciences and Pharmacy, College of Medicine, Health and Wellbeing, The University of Newcastle, Newcastle, NSW, Australia; ^5^ Transplantation Immunobiology Group, University of Sydney Central Clinical School, Charles Perkins Centre, Faculty of Medicine and Health, Sydney, NSW, Australia

**Keywords:** regulatory T cells, Foxp3, CTLA-4, PD-1, T cell proliferation, differential gene expression

## Abstract

Due to the intracellular expression of Foxp3 it is impossible to purify viable Foxp3^+^ cells on the basis of Foxp3 staining. Consequently CD4^+^Foxp3^+^ regulatory T cells (Tregs) in mice have mostly been characterized using CD4^+^CD25^+^ T cells or GFP-Foxp3 reporter T cells. However, these two populations cannot faithfully represent Tregs as the expression of CD25 and Foxp3 does not completely overlap and GFP^+^Foxp3^+^ reporter T cells have been reported to be functionally altered. The aim of this study was to characterize normal Tregs without separating Foxp3^+^ and Foxp3^-^ cells for the expression of the main functional molecules and proliferation behaviors by flow cytometry and to examine their gene expression characteristics through differential gene expression. Our data showed that the expressions of Foxp3, CD25, CTLA-4 (both intracellular and cell surface) and PD-1 was mostly confined to CD4^+^ T cells and the expression of Foxp3 did not completely overlap with the expression of CD25, CTLA-4 or PD-1. Despite higher levels of expression of the T cell inhibitory molecules CTLA-4 and PD-1, Tregs maintained higher levels of Ki-67 expression in the homeostatic state and had greater proliferation *in vivo* after allo-activation than Tconv. Differential gene expression analysis revealed that resting Tregs exhibited immune activation markers characteristic of activated Tconv. This is consistent with the flow data that the T cell activation markers CD25, CTLA-4, PD-1, and Ki-67 were much more strongly expressed by Tregs than Tconv in the homeostatic state.

## 1 Introduction

CD25 (1) and Foxp3 (2, 3) are the two most important biomarkers for identification of Tregs, with the former often used for Treg purification and the latter as the definitive Treg lineage marker (4). Here, we defined CD4^+^Foxp3^+^ T cells and CD4^+^Foxp3^-^ T cells as Tregs and Tconv respectively. As the α sub-unit of the IL-2 receptor, CD25 is uniquely expressed by Tregs but not Tconv in the homeostatic state and confers higher affinity of Tregs for IL-2 than Tconv (5–8). This is the foundation of low-level IL-2 treatment to specifically induce Treg but not Tconv expansion. There is, however, incomplete overlapping of Foxp3 and CD25 expression reported using transgenic green fluorescence protein (GFP) reporter mice (4), which indicated that CD25 is not specifically expressed on Tregs. As the GFP^+^Foxp3^+^ T cells were reported to be functionally altered due to transgenic manipulation (9, 10), we re-examined CD25 and Foxp3 expression and quantified the degree of their co-expression in normal mice of the same background as GFP mice. In addition to CD25 and Foxp3, the co-inhibitory molecule CTLA-4 was shown to be constitutively expressed in resting Tregs (11) and to control Treg-mediated immunosuppression (12). CTLA-4 is unusual in that although it is CTLA-4 on the cell surface that executes its function, most CTLA-4 molecules are located intracellularly. We were interested to examine the degree of co-expression between Foxp3 and CTLA-4 (intracellular and surface) and the correlation between intracellular and surface CTLA-4 expression. At the same time, the co-expression of Foxp3 and another co-inhibitory molecule PD-1 was also characterized. As CTLA-4 and PD-1 were mostly confined to the CD4^+^ T cell subset, their expressions by Tregs and Tconv in the homeostatic state and after skin transplantation were examined.

As it is impossible to purify viable Foxp3^+^ Tregs from normal animals, the proliferative behavior of Tregs is mostly assessed using CD4^+^CD25^+^ T cells which are different from CD4^+^Foxp3^+^ T cells because of incomplete overlap between the expression of CD25 and Foxp3. In addition, the purified CD4^+^Foxp3^+^ T cells from the transgenic GFP reporter mice were reported to be functionally altered (9, 10), and may not represent the actual proliferation of wild-type Tregs. Hence, we decided to examine the proliferation of Tregs and Tconv simultaneously without separating Foxp3^+^ and Foxp3^-^ cells in four scenarios: *in vitro* expansion of CD4^+^ T cells stimulated by allo-antigens assessed by carboxyfluorescein succinimidyl ester (CFSE); *in vivo* expansion of adoptively transferred CD4^+^ T cells assessed by CFSE; basally proliferating Tregs and Tconv in the homeostatic state assessed by Ki-67; *in vivo* expansion of endogenous Tregs and Tconv in skin transplant recipients assessed by Ki-67. Flow cytometric analysis of CFSE is one of the best ways to assess T cell proliferation, however, it is impossible to label T cells in normal mice with CFSE. Consequently, Ki-67 which is reported to have similar sensitivity as CFSE to detect antigen specific or allo-activated T cells (13) was used to quantify the proliferating cells in the third and fourth scenarios.

Foxp3, CD25, CTLA-4, and PD-1 are not only expressed by resting or naïve Tregs, but also are activation markers for Tconv (reviewed in (14)), which indicates that naïve Tregs share some molecular characteristics of activated Tconv. Hence, a more extensive examination of differential gene expression in resting Tregs and activated Tconv was compared with resting Tconv using the nCounter^®^ Immune Exhaustion Panel containing 785 genes covering immune activation and immunosuppression themes.

## 2 Materials and methods

### 2.1 Mice

C57BL/6 (CD45.2, H^-2b^) mice were purchased from the Central Animal House of the University of Newcastle, Australia. B6D2F1 (CD45.2, H^-2bd^) and congenic B6ly5.1 (CD45.1, H^-2b^) mice were purchased from the Animal Resource Centre in Western Australia. The only difference between B6ly5.1 mice and C57BL/6 mice are the CD45 isoform, with the former CD45.2 and the latter CD45.1. Monomeric red fluorescent protein (mRFP) mice (CD45.1, H^-2b^) were supplied by Associate Professor Alexandra Sharland from the University of Sydney. The mRFP mice (also known as Foxp3-IRES-mRFP mice) contained a knock-in gene faithfully co-expressing with Foxp3 in lymphocytes. All mice were male and from 6 to 12 weeks old. The mice were housed in Hunter Medical Research Institute. The ethics for use of animals was approved by the Animal Care and Ethics Committee of the University of Newcastle (A-2019-920).

### 2.2 Cell preparation

Cell isolation from lymph nodes and spleen were performed separately, but the same procedure was used for both. When preparing cells used for cell culture or adoptive transfer, the procedures were performed in a sterile class II biosafety cabinet. All the operations except centrifugation were performed on ice. The centrifuge was set at 450×g for 5 minutes at 4°C. The spleen or lymph nodes were transferred to a 70 µm strainer which was placed in a 100 mm petri-dish. The lymph nodes or spleen were then mashed with a 5ml syringe plunger in the 70µm strainer with cold phosphate buffered saline (PBS). After completion of the mashing, the cells were filtered into a 50 ml centrifuge tube through the 70µm strainer. The red cells were then lysed in lysis buffer for 5 minutes at room temperature. After washing, the cells were re-filtered through a 30-µm filter to remove cell clumps and re-suspended in PBS for cell counting.

### 2.3 Magnetic cell isolation

CD4^+^ T cells were isolated from pooled spleen and lymph node cells. The isolation was performed through magnetic negative selection using the CD4^+^ T Cell Isolation Kit (Miltenyi Biotec, #130-104-454) as per manufacturer’s instructions. Procedures were performed in a sterile class II biosafety cabinet. The purity of isolated CD4^+^ T cells was over 90% assessed by flow cytometry. The isolated CD4^+^ cells from B6ly5.1 mice were labelled with CFSE for *in vitro* cell culture and adoptive transfer experiments. The isolated CD4^+^ cells from mRFP mice were used for NanoString differential gene expression analysis.

### 2.4 CFSE labelling

All procedures minimised exposure to light. CFSE was diluted by adding 90µl DMSO to one vial of CFSE to make a 10 mM solution, which was further diluted 100 times to make a working solution before use. The cells were suspended in a concentration of 2×10^7^/ml in 2% FCS Dulbecco’s phosphate-buffered saline (DPBS). 50µl of diluted working concentration CFSE per 1ml of the suspended cells achieved a final concentration of 5 μM and the cells were incubated at room temperature for 5 minutes. The CFSE staining was quenched using 9 ml 10% FCS DPBS for 2 minutes. The CFSE stained cells were washed twice using 2% FCS DPBS and suspended in a suitable concentration for downstream work.

### 2.5 *In vitro* CD4^+^ T cell stimulation

CD4^+^T cells from the lymph nodes and spleen of each B6ly5.1 mouse were pooled and labelled with CFSE as responders and the irradiated splenocytes (15 Gray) from B6D2F1 mice as stimulators. The *in vitro* culture was performed as described in reference (15) for 3 days.

### 2.6 *In vivo* CD4^+^ T cell stimulation by adoptive T cell transfer

The CD4^+^ T cells from lymph nodes and spleens of all donor B6ly5.1 mice were pooled together before CFSE labelling. The cells were resuspended in sterile 0.9% injection saline and adoptively transferred to recipient B6D2F1 mice on a one-donor-to two-recipients basis. The injection volume for each mouse was 400 µl. After sufficient anaesthesia, the cells were injected to the recipient mice through the penile vein using a gauge 25 injection needle under the microscope with the dimmest microscopic light possible. After the injection was complete, the penis tip was gently pressed and pushed back into the prepuce using a sterile cotton bud. The mouse normally recovered in 2-5 minutes and then was transferred to a clean holding cage for further monitoring. The cells from lymph nodes and spleens were isolated and analysed separately at day 3.

### 2.7 Skin transplantation

C57BL/6 (H-2^b^) or mRFP (H-2^b^) mice were graft recipients and B6D2F1(H-2^bd^) mice were skin graft donors. 1×1 cm full-thickness tail skin was grafted on the back of the recipient mice. The skin graft was secured with medical grade glue and wrapped using adhesive bandage. The bandage was removed 7 days after transplantation.

### 2.8 Antibody staining for flow cytometric analysis

The antibodies and other reagents for flow cytometry work are listed in **Table 1**.

**Table 1:** Antibodies and viability dyes used in flow cytometry.

Viability or antibodies	Clone	Fluorophore	Company	Catalog	Optimal dilution
**CD45.1**	A20	BV605	BD Biosciences	747743	1/200
**CD3e**	500A2	R718	BD Biosciences	567303	1/200
**CD3e**	145-2C11	FITC	BD Biosciences	553061	1/200
**CD4**	GK1.5	BV786	BD Biosciences	563331	1/800
**CD8a**	53-6.7	APC-H7	BD Biosciences	560182	1/100
**CD25**	PC61	PE-cy7	BD Biosciences	552880	1/200
**Foxp3**	FJK-16S	PE	eBioscience	12-5773-8 2	1/200
**CD152 (CTLA-4)**	UC10-4F10-11	APC	BD Biosciences	564331	1/200
**CD279 (PD-1)**	RMP1-30	BV421	BD Biosciences	564071	1/200
**Ki-67**	B56	BUV395	BD Biosciences	564071	5µ/reaction
**Live/dead**	n/a	Fixable Viability Stain 510	BD Biosciences	564406	1/1000
**Live/dead**	n/a	LIVE/DEAD™ Fixable Near IR (780) Viability Kit (NIR)	eBioscience	L34994	1/1000

n/a, not applicable.

2.5 million cells were stained in a volume of 100µl in a FACS tube on ice. After Fc blocking, the cells were incubated with fluorochrome conjugated antibodies for cell surface molecules for 30 minutes with the addition of Brilliant Stain Buffer to make 100µl volume. After complete washing, the cells were incubated with the viability dye for another 30 minutes. The fluorochrome conjugated antibodies for intracellular molecules were stained for 30 minutes at room temperature after fixation and permeabilization using the Foxp3 Buffer Set as per manufacturer’s instructions.

All data acquisition was performed on the same BD LSR Fortessa X-20 cytometer and analysed using FlowJo 10.8.1. The expression level was assessed by median fluorescence intensity (MFI). The gating strategy for Foxp3, CD25, intracellular CTLA-4, surface CTLA-4, PD-1, and Ki-67 in skin transplant experiment is shown in **Supplementary Figure 1**. The gating strategies to gate Tregs and Tconv in *in vivo* (A and B) and *in vitro* (C and D) expansion is shown in **Supplementary Figure 2**.

### 2.9 Flow Cytometry Cell Sorting (FACS)

CD4^+^ T cells that had been isolated by magnetic cell isolation from mRFP reporter mice were then stained with viability dye for cell sorting for mRFP^+^Foxp3^+^ (Tregs) and mRFP^-^Foxp3^-^ (Tconv) cells by FACSAria III sorter. The mRFP was assessed in the PE channel. The gating strategy for Tregs and Tconv sorting are shown in **Supplementary Figures 2E, F**. The generated FACS data was re-analyzed in Flowjo and the gating strategy for Tregs and Tconv is shown in **Supplementary Figures 2G, H**.

### 2.10 Differential gene expression

The FACS sorted Tregs and Tconv were lysed in QIAGEN Buffer RLT Plus (# 1053393) with β-ME and stored at -80°C until RNA extraction. RNA was extracted using QIAGEN RNeasy Plus Mini Kit (Qiagen, Hilden, Germany) as per manufacturer’s instructions. Digital gene expression profiles were analysed using the nCounter Mouse Immune Exhaustion Panel (see gene list document and panel description in https://nanostring.com/products/ncounter-assays-panels/immunology/immune-exhaustion/), as per manufacturer’s instructions (NanoString Technologies).

The data was analysed using the Rosalind platform (https://www.rosalind.bio/). Differential gene expressions between T cell groups were determined as a p value<0.05 adjusted for false discovery rate using the Benjamini-Hochberg method, and a fold change (FC) of 1.5. The data was displayed as log_2_ FC [whereby ±1.5-FC difference is equivalent ±0.58 log_2_ FC].

### 2.11 Statistical analysis

The statistical analysis of the data in this project was performed using GraphPad Prism 9. Paired two-tailed t test and one-way ANOVA were used when comparing 2 and over 2 parameters, respectively, of samples from the same mouse. Likewise, unpaired two-tailed paired t test and one-way ANOVA were used when comparing 2 and over 2 parameters, respectively, of samples from different groups of mice. The data was shown in the figures as mean ± standard deviation (SD). The Log-rank (Mantel-Cox) test was used for graft survival analysis. It was considered significantly different if p < 0.05. For the range of values from p > 0.05, p ≤ 0.05, p ≤ 0.01, p ≤ 0.001 and p ≤ 0.0001 were shown as ns, *, **, *** and **** respectively. If the p value was near 0.05, the exact p value was shown. The data was shown in the figures as Mean ± SD. The differentially expressed genes in the differential gene expression experiments were screened using adjusted p < 0.05.

## 3 Results

### 3.1 The expressions of Foxp3, CD25, CTLA-4 and PD-1 is mostly confined to CD4^+^ T cells with their highest expression in CD4/CD8 double positive T cells in the normal homeostatic state

As shown in **Supplementary Figure 1G**, the frequency of CD4/CD8 double negative (DN) T cells in total T cells was low (less than 6%) and the frequency of DP T cells was much lower (less than 0.5% of total T cells). This is a contrast to CD4^+^ T cells ranging from 48.3% to 54.6% and CD8^+^ T cells ranging from 40.2% to 45.7%.

**Figure 1:**
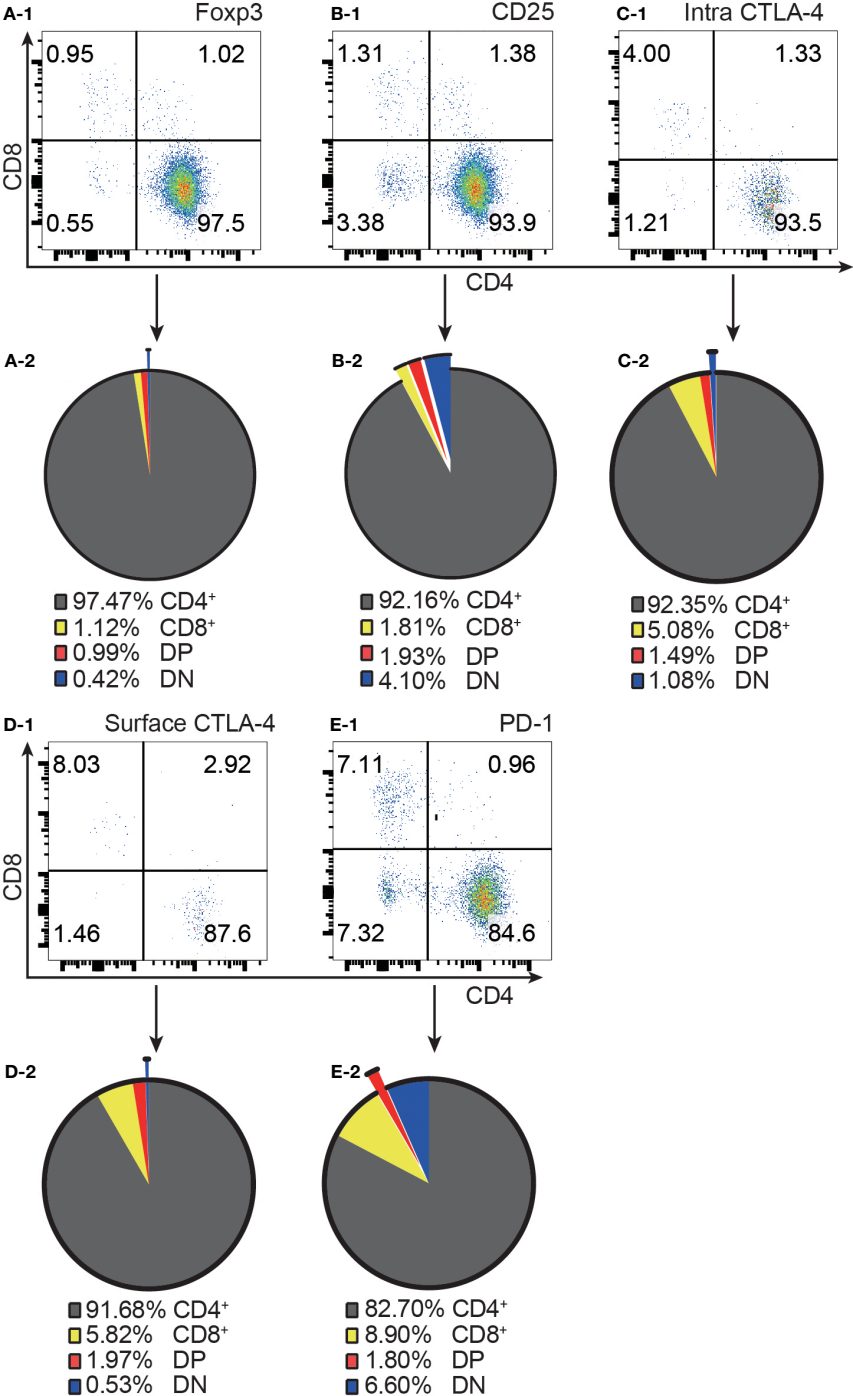
The distribution of Foxp3^+^, CD25^+^, intracellular CTLA-4^+^, surface CTLA-4^+^, and PD-1^+^ T cells in the four T cell subsets. **(A-1, B-1, C-1, D-1, E-1)** show representative flow cytometry data of the distribution of Foxp3^+^ T cells, CD25^+^ T cells, intracellular CTLA-4^+^ T cells, surface CTLA-4^+^ T cells, and PD-1^+^ T cells respectively in the four T cell subsets from normal C57BL/6 lymph nodes. The quadruple gates were set on CD3 positive Foxp3^+^ cells, CD25^+^ cells, intracellular CTLA-4^+^ cells, surface CTLA-4^+^ cells, and PD-1^+^ cells respectively. The pie charts of **(A-2, B-2, C-2, D-2, E-2)** show average percentage of the distribution of Foxp3^+^ T cells, CD25^+^ T cells, intracellular CTLA-4^+^ T cells, surface CTLA-4^+^ T cells, and PD-1^+^ T cells respectively in the four T cell subsets, (n=7). The data in spleen shows the same pattern.

**Figure 2:**
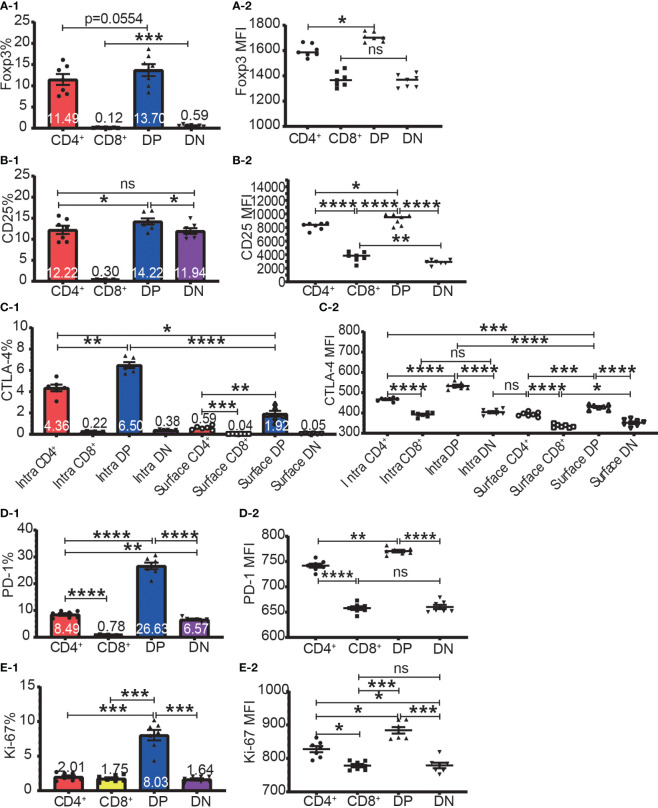
The expression of Foxp3, CD25, CTLA-4, PD-1, and Ki-67 in the four T cell subsets. The percentages of Foxp3^+^ cells, CD25^+^ cells, intracellular CTLA-4^+^ cells, surface CTLA-4^+^ cells, PD-1^+^ cells, and Ki-67^+^ cells in the four T cell subsets are shown in **(A-1, B-1, C-1, D-1, E-1)** respectively. The expression levels of Foxp3, CD25, intracellular CTLA-4 and surface, PD-1, and Ki-67 in the four T cell subsets are shown in **(A-2, B-2, C-2, D-2, E-2)** respectively. The data demonstrated is from lymph nodes of normal C57BL/6 mice and is shown as Mean ± SD (n = 7). Multiple-group comparison was performed using paired One-way ANOVA. For the range of values from p ≤ 0.05, p ≤ 0.01, p ≤ 0.001 and p ≤ 0.0001 were shown as *, **, *** and **** respectively. Ns, not significant.

Consistent with the published data from GFP mice (4), our results from normal C57BL/6 mice showed that approximately 97% of Foxp3^+^ T cells were confined to the CD4^+^ compartment (**Figure 1A-1, A-2**). It is important to point out that the distribution of Foxp3^+^ T cells into the CD8^+^ and DP compartments was at comparable levels, although the former was about 100 times the latter in cell number. Similar to Foxp3^+^ cells, the vast majority of CD25^+^, intracellular CTLA-4^+^, surface CTLA-4^+^ and PD-1^+^ T cells were also limited to CD4^+^ T cells (**Figure 1B-1–E-1, B-2–E-2**).

The highest frequencies of Foxp3^+^, CD25^+^, intracellular CTLA-4^+^, surface CTLA-4^+^, and PD-1^+^ T cells were always in DP T cell subset and their frequencies were slightly lower in CD4^+^ T cells and always lowest in CD8^+^ T cells (**Figure 2A-1–D-1**). The expression patterns of Foxp3 and CTLA-4 were the same in that their positive frequencies were higher in DP and CD4^+^ T cells than in DN and CD8^+^ T cells (**Figures 2A-1, C-1**). The expression patterns of CD25 and PD-1 were the same in that their positive frequencies were higher in DP, CD4^+^ and DN T cells than in CD8^+^ T cells (**Figure 2B-1, D-1**). The frequencies of Foxp3^+^, CD25^+^, intracellular CTLA-4^+^, surface CTLA-4^+^, and PD-1^+^ cells in CD8^+^ T cell subsets were all very low. For example, the Foxp3^+^ cell percentage in CD8^+^ T cells was less than 0.25% and as low as 0.045%, which is virtually undetectable.

**Figure 3:**
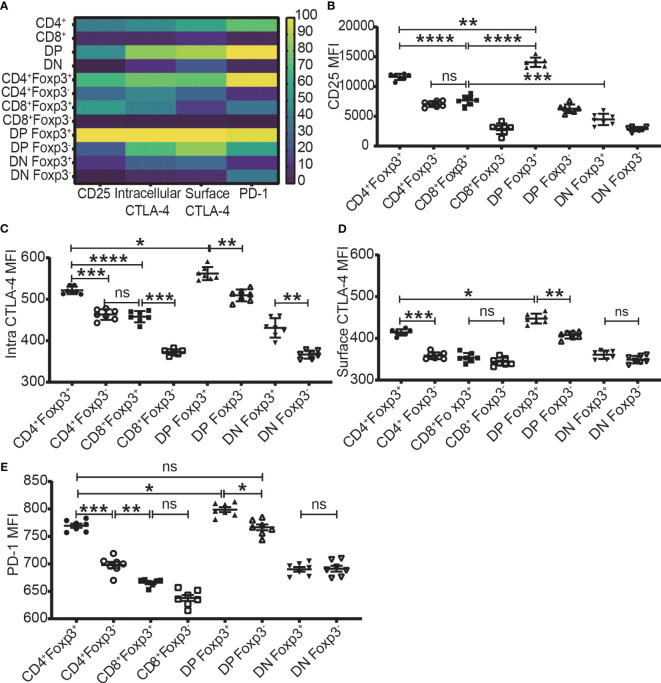
The expression levels of CD25, CTLA-4, and PD-1 in the four T cell subsets and their Foxp3^+^ and Foxp3^-^ subpopulations. **(A)** shows a heatmap of the expression levels of CD25, intracellular CTLA-4, surface CTLA-4, and PD-1 in the four T cell subsets corresponding to A-2 to E-2 in **Figure 2** and in their Foxp3^+^ and Foxp3^-^ subpopulations corresponding B to E of this Figure. The values of each parameter were normalized across T cell subsets and their subpopulations, but not across different parameters, meaning that comparison can only be performed on one parameter between T cell subsets (vertically) and their subpopulations but cannot performed on multiple parameters in one subset (horizontally). The normalized score is between 1 to 100. **(B–E)** show the expression level of CD25, intracellular CTLA-4, surface CTLA-4, and PD-1 in Foxp3^+^ and Foxp3^-^ subpopulations of the four T cell subsets. The data demonstrated is from lymph nodes of normal C57BL/6 mice and is shown as Mean ± SD (n = 7). Multiple-group comparison was performed by paired One-way ANOVA. The data in spleen shows a similar pattern. For the range of values from p ≤ 0.05, p ≤ 0.01, p ≤ 0.001 and p ≤ 0.0001 were shown as *, **, *** and **** respectively. Ns, not significant.

The expression level of Foxp3, CD25, intracellular CTLA-4, surface CTLA-4, and PD-1 were highest in DP T cells, slightly lower in CD4^+^ T cells, and lowest in CD8^+^ and DN T cells (**Figures 2A-2–D-2**; **Figure 3A**). When it came to the Foxp3^+^ and Foxp3^-^ subpopulations of the four T cell subsets, the expression levels of these molecules were always higher in Foxp3^+^ cells than Foxp3^-^ cells with the highest level in Foxp3^+^ DP cells and slightly lower in Foxp3^+^CD4^+^ T cells (**Figure 3**).

### 3.2 The expression of Foxp3 did not completely overlap with the expression of CD25

**Figure 4:**
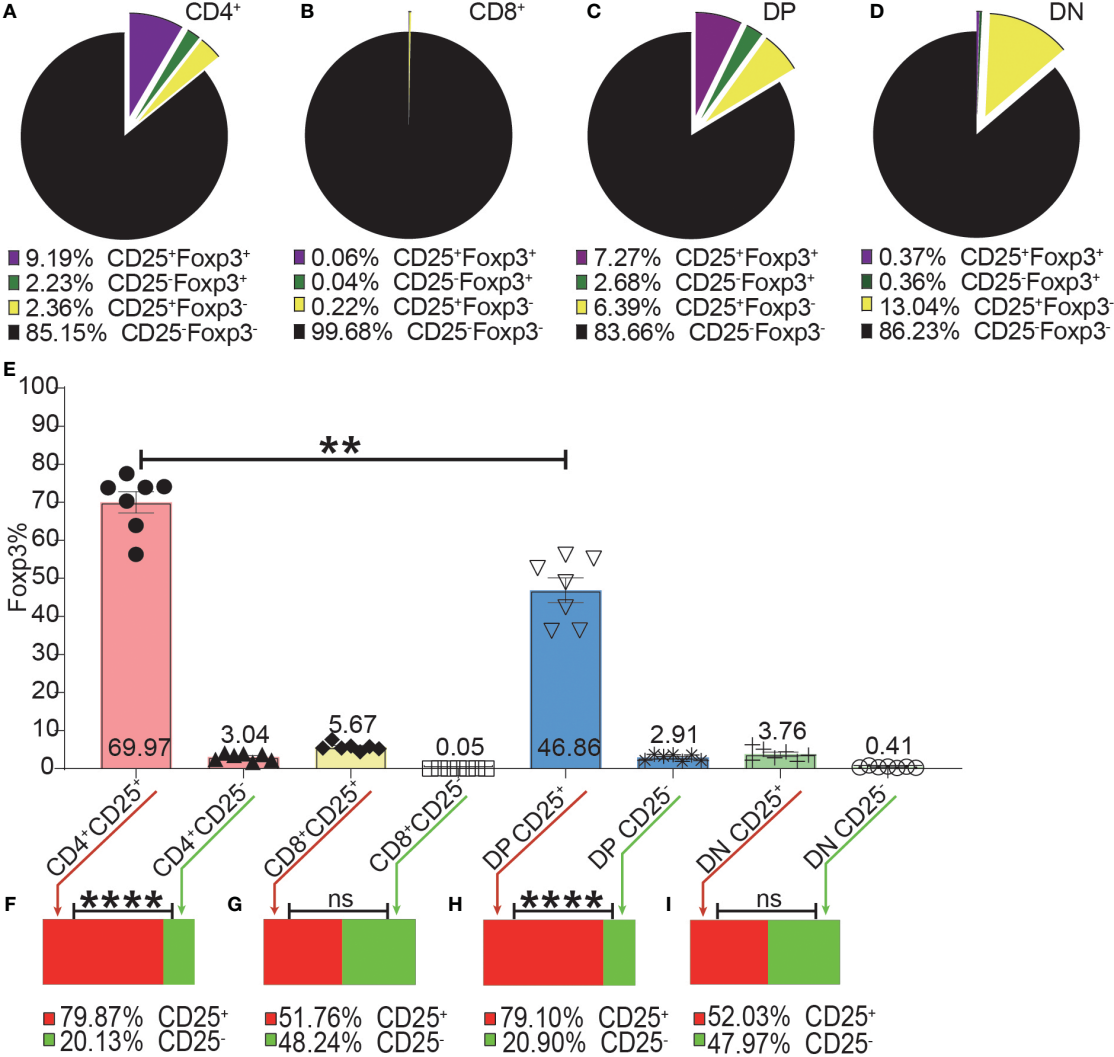
The incomplete overlapping between Foxp3 and CD25 expression in the four T cell subsets. The pie charts of **(A–D)** show the proportions of CD25^+^Foxp3^+^, CD25^-^Foxp3^+^, CD25^+^Foxp3^-^, CD25^-^Foxp3^-^ in the four T cell subsets. The data shows the Mean from 7 mice. **(E)** shows the frequencies of Foxp3^+^ cells in the CD25^+^ and CD25^-^ subpopulations of the four T cell subsets, which were generated by placing a CD25 gate on each of the four T cell subsets and then placing a Foxp3 gate on each of the generated CD25^+^ and CD25^-^ subpopulations. The bar charts of **(F–I)** show the distribution of Foxp3^+^ cells of each T cell subset into their CD25^+^ and CD25^-^ subpopulations, which was generated by placing a Foxp3 gate on each of the four T cell subsets and then placing a CD25 gate on each of the generated Foxp3^+^ subpopulations. The data in **(E)** is shown as Mean ± SD and the data in bar charts is shown as Mean, n = 7. The data demonstrated is from lymph nodes of normal C57BL/6 mice. The data in spleen shows similar pattern. For the range of values from p ≤ 0.01 and p ≤ 0.001 were shown as ** and *** respectively. Ns, not significant.

As shown in **Figure 4A–D**, the expressions of CD25 and Foxp3 did not completely overlap in any of the four T cell subsets. The overlap of CD25 and Foxp3 expression in CD8^+^ and DN T cells was poorer than in CD4^+^ and DP T cells. The highest frequency of Foxp3^+^ cells in CD25^+^ cells was in the CD4^+^ T cell subset and then lower in DP T cells while the percentages of Foxp3^+^ cells in CD25 positive CD8^+^ and DN T cells were very low (**Figure 4E**). These data indicated that about 30%, 50%, 80%, and 97% of CD25^+^ cells were Foxp3 negative in CD4^+^, DP, CD8^+^ and DN T cells respectively. This suggested that Treg purification by CD25 would inevitably cause dramatic contamination of CD25^+^ Tconv which would be preferably expanded by low-level IL-2 treatment. About 80% of Foxp3^+^ cells in CD4^+^ T cells and DP T cells were CD25 positive while these percentages in CD8^+^ and DN T cells were about 50% (**Figures 4F–I**). These data showed that there was also a sizeable proportion of Tregs that did not express CD25 and would not benefit from low-level IL-2 treatment.

### 3.3 The expression of CTLA-4 or PD-1 did not completely overlap with the expression of Foxp3 and CD25

**Figure 5:**
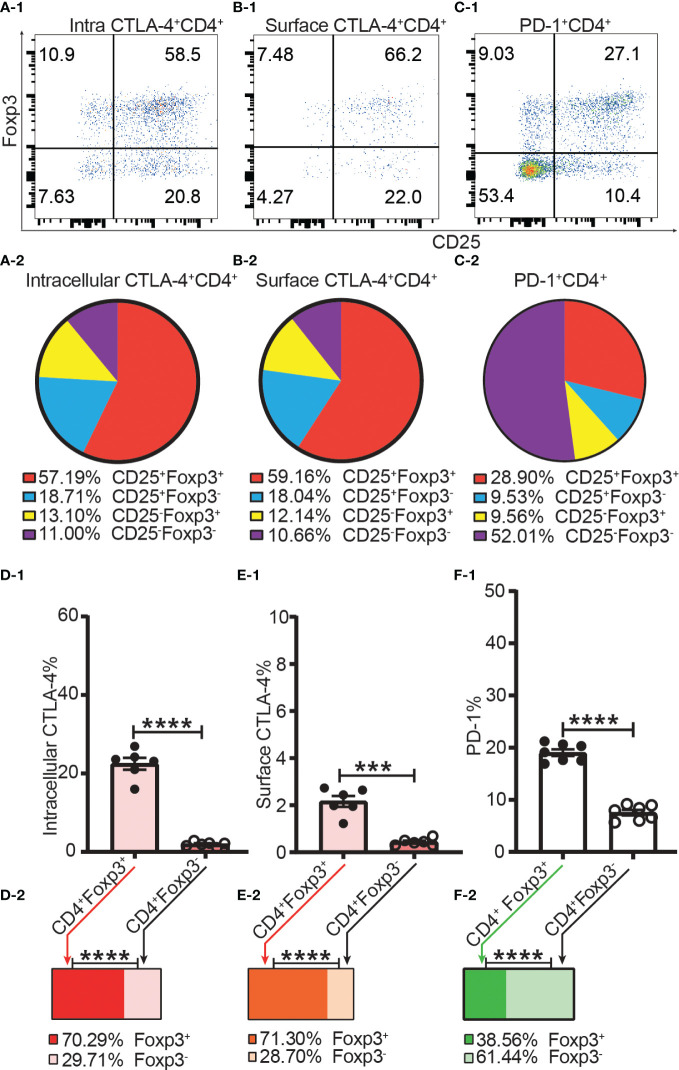
The incomplete overlapping between the expression of Foxp3 and the expression of CTLA-4 and PD-1 in CD4^+^ T cells. Dot plots **(A-1)**, **(B-1)**, and **(C-1)** show the distribution of intracellular CTLA-4^+^CD4^+^ T cells, surface CTLA-4^+^CD4^+^ T cells and PD-1^+^CD4^+^ T cells respectively into CD25^+^Foxp3^+^, CD25^+^Foxp3^-^, CD25^-^Foxp3^+^, and CD25^-^Foxp3^-^ compartments of CD4^+^ T cells. Pie charts of **(A-2)**, **(B-2)**, and **(C-2)** shows the Mean of **(A-1)**, **(B-1)**, and **(C-1)** respectively (n = 7). **(D-1)**, **(E-1)**, and **(F-1)** show the percentage of intracellular CTLA-4^+^, surface CTLA-4^+^, and PD-1^+^ cells in Tregs and Tconv respectively. The bar charts of (D-2), **(E-2)**, and **(F-2)** show the distribution of intracellular CTLA-4^+^CD4^+^ cells, surface CTLA-4^+^CD4^+^ cells, and PD-1^+^CD4^+^ cells respectively into Treg and Tconv compartments. The data in A-2 to C-2 and D-2 to F-2 are shown as Mean and the data in D-1 to F-1 are shown as Mean ± SD, n =7. The data demonstrated is from lymph nodes of normal C57BL/6 mice and the data in spleen has the similar pattern. For the range of values from p ≤ 0.001 and p ≤ 0.0001 were shown as *** and **** respectively.

As Foxp3, CD25, CTLA-4, and PD-1 were mostly expressed in the CD4^+^ T cell subset, we focused our analysis on CD4^+^ T cells. Not all intracellular CTLA-4^+^, surface CTLA-4^+^ or PD-1^+^ T cells were CD25 or Foxp3 positive, and a significant proportion of them were CD25 and Foxp3 double negative (**Figures 5A-1–C-1, A-2–C-2**), which suggested that at least part of CTLA-4 and PD-1 might work independently of both Foxp3 and CD25.

The frequencies of intracellular CTLA-4^+^, surface CTLA-4^+^ and PD-1^+^ were all higher in Tregs than in Tconv (**Figures 5D-1–F-1**). Our data showed only about 20% of Tregs expressed CTLA-4 intracellularly, and only about 2% of Tregs expressed CTLA-4 on the cell surface (**Figures 5D-1, E-1**). In other words, although it was shown that CTLA-4 controls Treg function (12), only a small proportion of Tregs expressed CTLA-4 intracellularly and a tiny fraction of Tregs expressed CTLA-4 on the cell surface. In addition, this also suggested that not all Tregs expressing CTLA-4 intracellularly expressed CTLA-4 on the cell surface at the same time. The distribution of intracellular CTLA-4^+^ cells and surface CTLA-4^+^ cells was greater in the Treg than in the Tconv compartment (**Figure 5D-2, E-2**) while more PD-1^+^ T cells were distributed in the Tconv than in the Treg compartment (**Figure 5F-2**), which showed that CTLA-4 and PD-1 are constitutively expressed by a small proportion of both Tregs and Tconv in the homeostatic state.

### 3.4 Dynamics of expression of Foxp3, CD25, CTLA-4 and PD-1 after skin transplantation in mice

As will be discussed in 3.5, T cells were not activated at day 3 but had been activated at day 7 post-transplantation.

**Figure 6:**
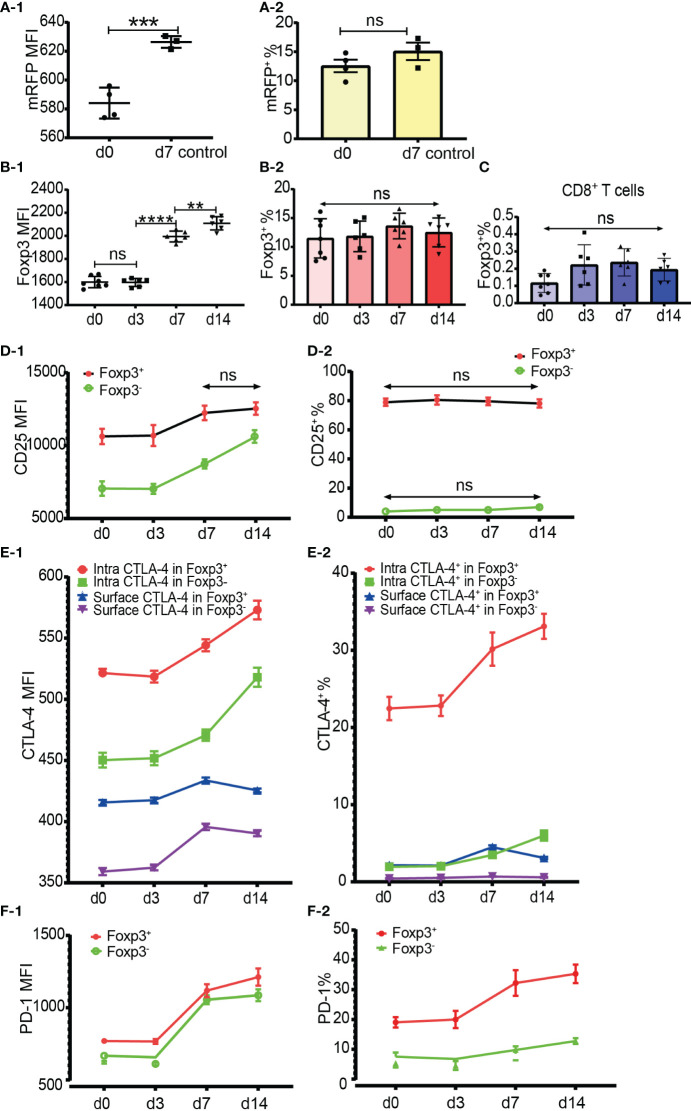
Dynamics of Foxp3, CD25, CTLA-4 and PD-1 expression after skin transplantation. **(A-1, A-2)** show mRFP expression level and percentage positive of mRFP^+^ T cells respectively in CD4^+^ T cells at day 0 (non-transplanted) and day 7 from draining lymph nodes. The data was displayed as Mean ± SD, n =4 at day 0 (non-treated) and n = 3 at day 7. **(B-1, B-2)** show Foxp3 expression level and percentage positive of Foxp3^+^ T cells respectively in CD4^+^ T cells at day 0, 3, 7 and 14. **(C)** shows the percentage positive of CD8^+^Foxp3^+^ T cells at day 0, 3, 7, and 14. **(D-1, D-2)** show CD25 expression level and percentage of CD25^+^ cells in Tregs and Tconv at day 0, 3, 7, and 14. **(E-1, E-2)** show CTLA-4 expression level and percentage positive of CTLA-4^+^ cells in Tregs and Tconv at day 0, 3, 7, and 14. **(F-1, F-2)** show PD-1 expression level and percentage positive of PD-1^+^ cells in Tregs and Tconv at day 0, 3, 7, and 14. The data is displayed as Mean ± SD, n =7 at day 0, n = 6 at day 3, 7, and 14. Two-group comparison was performed using unpaired t test and multiple-group comparison was performed using unpaired one-way ANOVA. For the range of values from p ≤ 0.01, p ≤ 0.001 and p ≤ 0.0001 were shown as **, *** and **** respectively. Ns, not significant.

To examine whether the percentage of Foxp3^+^ cells increased after allo-activation, we employed both mRFP-Foxp3 reporter mice and normal C57BL/6 mice as recipients of B6D2F1 skin transplants. The expression levels of mRFP and Foxp3 both had been significantly upregulated at day 7 after skin transplantation (**Figures 6A-1, B-1**). However, the percentage of mRFP^+^ cells in CD4^+^ T cells at day 7 (only day 7 timepoint was observed for mRFP reporter T cells) and the percentage of Foxp3^+^ cells in CD4^+^ T cells at day 7 and day 14 did not significantly change (**Figures 6A-2, B-2**). As Foxp3 positive CD8^+^ Tregs were also reported (16, 17), we then checked Foxp3 expression in CD8^+^ T cells after allo-activation and found that the low percentage of Foxp3^+^ cells in CD8^+^ T cells did not significantly increase (**Figure 6C**).

It is generally accepted that CD25 is upregulated in Tconv after activation and that it is not reliable to be used as a sorting marker for Tregs, especially after CD4^+^CD25^+^ T cell expansion. Our data showed that although the CD25 expression level increased, the percentage of CD25^+^CD4^+^ T cells did not significantly increase at day 7 and day 14 after transplantation (data not shown). When explored further, same as in CD4^+^ T cells, although the CD25 expression level increased, the percentage of CD25^+^ cells in Tregs and Tconv also did not significantly increase at day 7 and day 14 (**Figures 6D-1, D-2**). These data suggested that CD25 expression could not be induced from CD25 negative cells, at least in the model of mouse skin transplantation.

The expression level and percentage positive of CTLA-4 (both intracellular and surface) and PD-1 had been upregulated at day 7 (**Figures 6E-1, E-2, F-1, F-2**). Like resting T cells, the intracellular CTLA-4 level was higher than surface CTLA-4 in both Tregs and Tconv and increased after T cell activation (**Figure 6E-1**). The expression of PD-1 and intracellular CTLA-4 in Tregs and Tconv was significantly upregulated at day 7 and day 14 timepoints (**Figures 6E-1, F-1**). Although slightly increased, the surface CTLA-4 expression level and percentage positive in both Tregs and Tconv were always maintained at a low level (**Figures 6E-1, E-2**). Compared with the day 7 timepoint, the expression level of surface CTLA-4 slightly dropped at day 14 (**Figure 6E-1**). The percentages of intracellular CTLA-4^+^ and PD-1^+^ cells both in Tregs and Tconv increased from day 7 (**Figures 6E-2, F-2**), which indicated that CTLA-4 and PD-1 expression could be induced from CTLA-4^-^ cells and PD-1^-^ cells respectively after T cell activation.

### 3.5 Foxp3^+^ T cells maintained a higher ratio of proliferating cells than Foxp3^-^ T cells in the homeostatic state and after allo-activation

**Figure 7:**
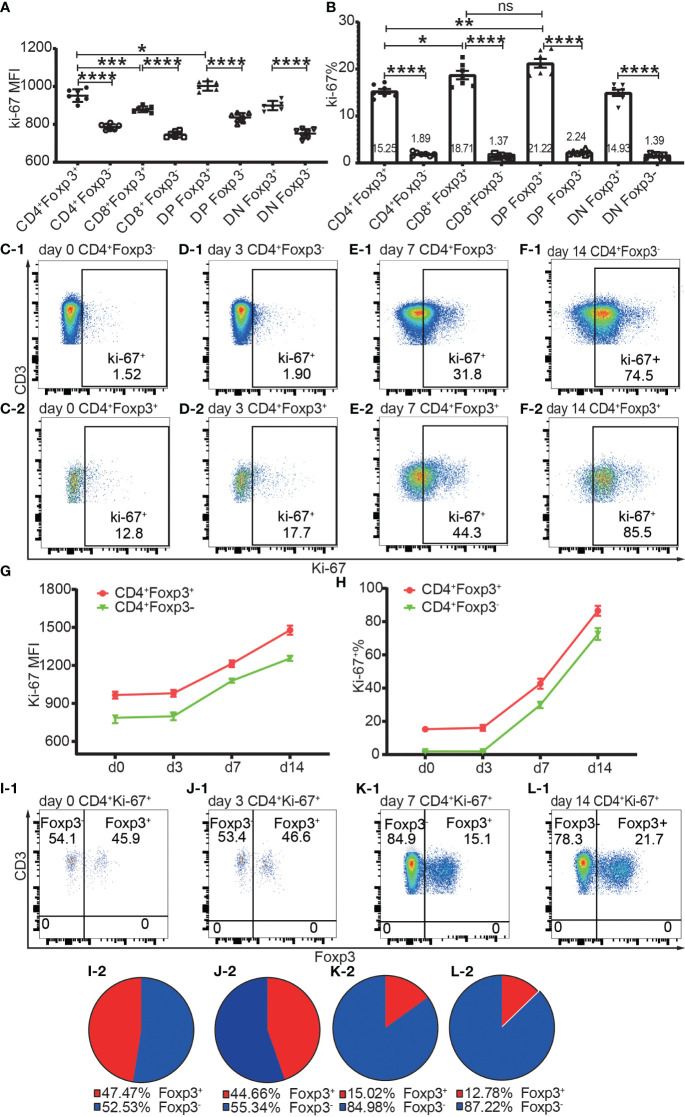
The expression of Ki-67 in Foxp3^+^ and Foxp3^-^ T subpopulations in the homeostatic state and after allo-activation. The proliferation was assessed by Ki-67 expression and the data is from draining lymph nodes. **(A. B)** show the expression level of Ki-67 and percentage of Ki-67^+^ cells respectively in Foxp3^+^ and Foxp3^-^ subpopulations of the four T cell subsets at day 0 (non-transplanted). The data is shown as Mean ± SD, n = 7. **(C-1, D-1, E-1, F-1)** show representative dot plots of percentages of Ki-67^+^ cells in Tconv at day 0, 3, 7, and 14 respectively. **(C-2, D-2, E-2, F-2)** show representative dot plots of percentages of Ki-67^+^ cells in Tregs at day 0, 3, 7, and 14 respectively. **(G, H)** show the expression levels of Ki-67 and percentages of Ki-67^+^ cells in Tconv and Tregs at day 0, 3, 7, and 14. The data is shown as Mean ± SD, n =7 at day 0, n = 6 at day 3, 7, and 14. The dot plots of **(I-1, J-1, K-1, L-1)** show the distribution of Ki-67^+^CD4^+^ T cells into Tconv and Treg compartments at day 0, 3, 7, and 14. The pie charts of **(I-2, J-2, K-2, L-2)** show the Mean of **(I-1, J-1, K-1, L-1)** respectively, n =7 at day 0, n = 6 at day 3, 7, and 14. For the range of values from p ≤ 0.05, p ≤ 0.01, p ≤ 0.001 and p ≤ 0.0001 were shown as *, **, *** and **** respectively.

Proliferation was assessed by Ki-67 and Ki-67^+^ T cells were defined as proliferating and activated T cells. Both Foxp3^+^ cells and Foxp3^-^ cells of the four T cell subsets maintained a low level of Ki-67 expression and low ratio of Ki-67^+^ cells in the homeostatic state (**Figures 7A, B**). The Ki-67 expression level and Ki-67^+^ percentage were both higher in Foxp3^+^ cells than in Foxp3^-^ cells (**Figures 7A, B**). The average ratios of proliferation in CD4^+^Foxp3^+^ Tregs and CD4^+^Foxp3^-^ Tconv were 15.25% and 1.89% respectively (**Figure 7B**).

The Ki-67 expression level and Ki-67^+^ percentage in Tregs and Tconv did not significantly increase at day 3 but had increased at day 7 (**Figures 7C-1–F-1, C-2–F-2, G, H**), which indicated that T cells began to proliferate at a timepoint between day 3 and day 7 in the murine skin transplant model. The endogenously expanded Tregs maintained a slightly higher proliferating percentage than in Tconv after allo-activation at day 7 and day 14 (**Figure 7G, H**).

About half of the proliferating cells in CD4^+^ T cells were distributed in the Foxp3^+^ compartment before allo-activation at day 0 and day 3 (**Figures 7I-1, I-2, J-1, J-2**), which suggested that the ratio of functioning self-reactive Tregs and Tconv might be about the same number in the normal homeostatic state. This is in contrast to the assumption that the 5% to 10% Tregs counterbalance the majority of Tconv (18). We then calculated the ratio of proliferating allo-reactive Treg/Tconv in the Ki-67^+^CD4^+^ T cells at day 7 and day 14. The average ratios at day 7 and day 14 were 1:5.92 and 1: 6.98, respectively (**Figures 7K-1, K-2, L-1, L-2**). This indicated that allo-reactive Tregs were dominated by allo-reactive Tconv in transplant rejection and about 5 to 6-fold more of activated allo-reactive Tregs might be required to achieve transplant tolerance.

### 3.6 Tregs and Tconv exhibit different proliferation characteristics *in vivo* and *in vitro*

The *in vivo* and *in vitro* proliferation of Tregs and Tconv was assessed by flow cytometric analysis of CFSE at day 3 after adoptive transfer and cell culture. Different application settings were used for the flow cytometric analysis of *in vitro* and *in vivo* proliferation. The gating strategies for Tregs and Tconv *in vivo* and *in vitro* are shown in **Supplementary Figures 2A–D**.

**Figure 8:**
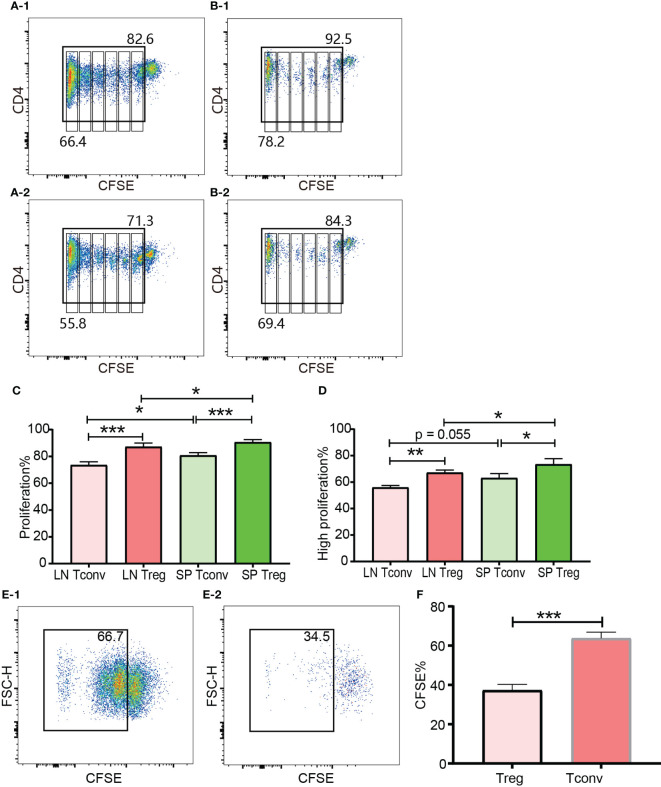
*In vivo* and *in vitro* expansion of Tregs and Tconv at day 3. The proliferation was assessed by CFSE. **(A-1, B-1)** show the proliferation of donor Tconv and Tregs respectively in recipient spleen. **(A-2), (B-2)** show the proliferation of donor Tconv and Tregs respectively in recipient lymph nodes. The rightmost column was non-proliferating cells, and the leftmost column was defined as highly proliferating cells. Six cell division peaks can be identified in both of Tconv and Tregs. **(C)** shows the percentages of proliferating Tregs and Tconv. **(D)** shows the percentages of highly proliferating Tregs and Tconv. The data was shown as Mean ± SD, n = 3. The comparison between two groups was performed by paired t test. **(E-1, E-2)** show the proliferation of Tconv and Tregs *in vitro* at day 3. Two ambiguous division peaks can be identified in both of Tconv and Tregs. All these dividing cells were defined as proliferating cells. **(F)** is comparison of the percentage of proliferating Tregs and Tconv *in vitro*. The data in **(F)** was displayed as Mean ± SD, n = 3. The comparison between two groups was performed by paired t test. For the range of values from p ≤ 0.05, p ≤ 0.01 and p ≤ 0.001 were shown as *, ** and *** respectively.

In the *in vivo* expansion model, donor cells consisting of pooled B6Ly5.1 CD4^+^ T cells from lymph nodes and spleen were injected into B6D2F1 mice on a one-donor-to-two-recipient basis. Both donor Tconv and Tregs proliferated after encountering allo-antigens in lymph nodes and spleen of the cell recipient mice (**Figures 8A-1, A-2, B-1, B-2**). Among the expanded cells, the leftmost peak was defined as highly proliferating cells. There were as many as 6 peaks present, which meant that part of the donor cells had divided 6 times and the cell number expanded by 2^6^ = 64 times. The vigorous *in vivo* T cell expansion indicated that the cells were not functionally compromised by the *in vitro* operations to prepare cells for injection including red blood cell lysis and magnetic selection. The percentage of donor CD4^+^ T cells accounted for 10.40% to 19.29% (14.35 ± 3.71) of total CD4^+^ T cells from recipient lymph nodes. This percentage range in the spleen was from 24.8% to 39.55% (31.14 ± 5.44). The proportion of donor cells in spleen was significantly higher than in lymph nodes. This might be, at least partially, due to the fact that in preparation of donor cells for injection more cells were generated from the spleen than from the lymph nodes, and these cells were inclined to recirculate to the lymphoid organs from which they were derived. Another possibility was that the cells were adoptively transferred via the blood stream and would reach the spleen earlier than the lymph nodes. Whether in the lymph nodes or spleen, donor Tregs maintained a slightly higher percentage of proliferation than donor Tconv (**Figure 8C, D**). The higher ratio of proliferating cells in Tregs than in Tconv after allo-activation was consistent with the data assessed using Ki-67 in skin transplant models discussed in subheading 3.5.

The cells for *in vitro* cell culture and *in vivo* adoptive transfer underwent the same preparations (e.g., physical disruption of the lymph nodes and spleen for cell isolation, blood cell lysis, magnetic CD4^+^ isolation, and CFSE labelling). Although published protocols were closely followed both Tregs and Tconv proliferated very poorly in the presence of irradiated B6D2F1 allo-splenocytes (**Figures 8E-1, E-2**). Except the left most column (probably background) there were two poorly defined dividing peaks present, which meant that a small proportion of T cells had expanded as many as 4 times. This was in contrast to the *in vivo* proliferation of Tregs and Tconv and indicated that the *in vitro* behaviour of Tregs and Tconv was not representative of their behaviour *in vivo*, which was probably because it was difficult to perfectly mimic the *in vivo* conditions *in vitro*. In regard to the relative proliferation of Tregs compared with Tconv, the proliferating percentage in Tregs was significantly less *in vitro* (**Figure 8F**).

### 3.7 Differential gene expression revealed that naïve Tregs had similar molecular characteristics to activated Tconv

**Figure 9:**
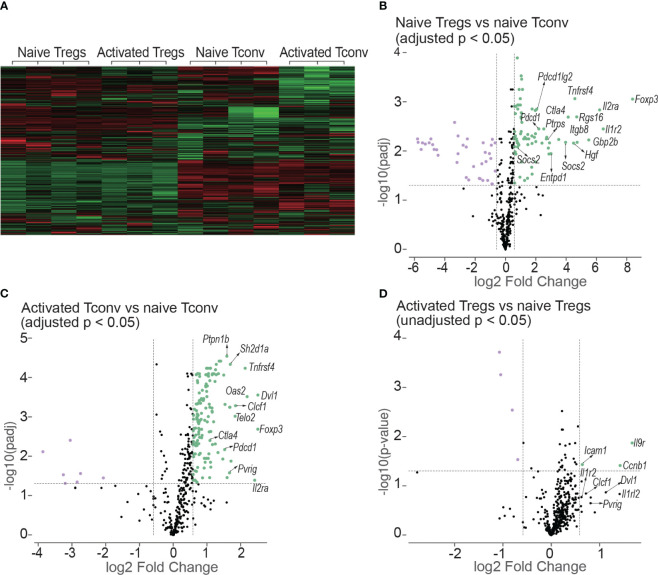
Heatmap and volcano plots in differential gene expression analysis. **(A)** shows the heatmap of normalized values of all the 14 samples: naive Tregs (n =4); naive Tconv (n = 4); activated Tregs (n = 3); activated Tconv (n = 3). The volcano plots above the dotted horizontal line show the differentially expressed genes between naïve Tregs and naïve Tconv **(B)**; between activated Tconv and naïve Tconv **(C)**; between activated Tregs and naïve Tregs **(D)**. The differentially expressed genes were screened using adjusted p < 0.05 in **(B, C)** but using unadjusted p < 0.05 as no genes were screened using adjusted p < 0.05. The vertical lines represent the 1.5-fold change.

Tregs (CD4^+^mRFP^+^Foxp3^+^) and Tconv (CD4^+^mRFP^-^Foxp3^-^) sorted from non-transplanted mRFP mice were termed “naïve Tregs” (n = 4) and “naïve Tconv” (n = 4) respectively. As the flow cytometry data confirmed that T cells had been activated at day 7 after transplantation, Tregs and Tconv sorted from allogeneic skin recipient mRFP mice at day 7 were termed “activated Tregs” (n = 3) and “activated Tconv” (n = 3) respectively. The heatmap of all the 14 samples is shown in **Figure 9A**.

The differentially expressed genes between naïve Tregs and naïve Tconv screened by adjusted p < 0.05 are shown in **Figure 9B** and **Supplementary Data 1**. Consistent with the flow cytometry data the expression of CD25, CTLA-4, and PD-1 encoding genes *Il2ra*, *Ctla4*, and *Pdcd1* were all higher in Tregs than in Tconv. The top 5 gene sets for the differentially expressed genes were IL-10, T cell checkpoint, T cell exhaustion, IL-2, and CTLA-4. The expressions of co-stimulatory and co-inhibitory molecule-encoding genes was all higher in naïve Tregs than in naïve Tconv. The co-stimulatory molecule-encoding genes also included those responsible for activation-induced molecules such as ICOS and LAG3. Not only genes for co-inhibitory molecules (e.g., *Ctla4* and *Pdcd1*) and immunosuppressive cytokines (e.g., *Il10*), but also genes for their ligands or receptors (e.g., *Cd86, Cd274, Pdcd1lg2* and *Il10rb*) were higher in Tregs than in Tconv. The gene sets were generally classified into two groups. The first group was defined as T cell negative regulation related genes which contained co-inhibitory, anergy, senescence & quiescence, apoptosis, and immunosuppressive cytokine- associated genes. Except for immunosuppressive cytokines, all these mechanisms are proposed to be involved in negative regulation of conventional T cells and maintaining peripheral tolerance (reviewed in (19)). For the immunosuppressive cytokines such as IL-10 and TGF-β, they are typically expressed in Tregs and important for Treg-mediated immunosuppression. The second group was defined as T cell activation related gene sets which contains T cell activation (TCR, co-stimulatory and inflammatory cytokine), intracellular signal transduction molecules (PI3K-AKT, JAK/STAT, NF-κB, MAKP, Notch and mTOR), and cell cycle related genes. In addition, the genes for suppressor of cytokines such as *Cish, Socs2*, and *Socs3* were expressed higher in Tregs than in Tconv.

The differentially expressed genes between activated Tconv and naïve Tconv (adjusted p < 0.05) were shown in **Figure 9C** and **Supplementary Data 2**. Some typical T cell activation marker encoding genes such as *Cd69* and *Cd101* were upregulated and the gene for CD62L (*Sell*) was downregulated in Tconv at day 7 after skin transplantation, which indicated that consistent with the flow cytometry data assessed by Ki-67, T cells had been allo-activated at this timepoint. From the gene sets of antigen presentation, TCR and T cell checkpoint, the genes encoding MHC molecules (e.g., *H2-d1, H2-k1, H2-m3, H2-t23*), TCR and its activation associated molecules (e.g., Cd3d, *Trat1, Trbc1/2, Trdv2-1/2-2, Zap70*), activation-induced molecules (e.g., *Icos, Tnfrsf14, Tnfrsf18, Tnfrsf4*), and co-inhibitory molecules (e.g., *Ctla4* and *Pdcd1*) as well as ligands of PD-1 (*Cd274* and *Pdcd1lg2*) were upregulated. In addition, the gene expression of major functional molecules of various intracellular signal transduction pathways including AP-1, mTOR, Notch, MARP, JAK/STAT, NF-κB, and PI3K-AKT increased.

For the comparison between activated Tregs and naïve Tregs, no differentially expressed genes were obtained by adjusted p < 0.05. Instead, the differentially expressed genes were screened using unadjusted p value < 0.05 as shown in **Figure 9D** and **Supplementary Data 3**. However. the genes encoding molecules for T cell activation and T cell checkpoint and intracellular signal transduction present in activated Tconv were not present in Tregs after allo-activation.

## 4 Discussion

### 4.1 Foxp3 and CD25 expression in CD8^+^ T cells

The percentages of both Foxp3^+^ and CD25^+^ cells were very low and even undetectable in CD8^+^ T cells. These percentages did not significantly increase after allo-activation. In addition, overlap of Foxp3 and CD25 expression in CD8^+^ T cells was very poor. Although CD8^+^CD25^+^ (20, 21), CD8^+^Foxp3^+^ (22), and CD8^+^CD25^+^Foxp3^+^ (23, 24) T cells were shown to be immunosuppressive and have been proposed as CD8^+^ Tregs, these populations are numerically small and are likely to be more challenging than CD4^+^ Tregs to translate to clinical therapy.

### 4.2 Peripheral DP T cells were characteristic of CD4^+^ T cells but with higher basal Ki-67 expression

DP T cells have been extensively studied during their development in the thymus but are less well-characterised in the periphery. For thymic DP cells, Foxp3 (2, 4) and CD25 (25) have not been detected. Our data showed that both Foxp3 and CD25 expression were higher in peripheral DP T cells than in other T cell subsets, indicating that the peripheral DP T cells are different from those immature thymic DP T cells. Not only Foxp3 and CD25, but also the expressions of intracellular CTLA-4, surface CTLA-4, and PD-1 as well as the proliferation marker Ki-67 were highest by peripheral DP T cells among the four T cell subsets (**Figure 2**). Hence, we speculated that peripheral DP T cells might be an activated cell population. As DP T cells were reported to dramatically increase in certain diseases (reviewed in (26)) and *in vitro* induced CD4^+^CD8^+^Foxp3^+^ from CD4^+^Foxp3^+^ T cells were more immunosuppressive (27), this unique T cell subset deserves more attention either for diagnosis value or for therapeutic purposes.

### 4.3 The incomplete overlapping between the expression of Foxp3 and CD25

Although it is widely accepted that Foxp3 has been the best marker for Tregs, the definition of regulatory T cells has been inconsistent. For example, the term “Tregs” is interchangeably used for CD4^+^Foxp3^+^ T cells, CD4^+^CD25^+^Foxp3^+^ T cells, and CD4^+^CD25^+^ T cells. This presupposes the complete co-expression of CD25 and Foxp3 in CD4^+^ T cells. The incomplete overlap of the expression of CD25 and Foxp3 from our data indicated that it might present problems for both adoptive Treg therapy and low-level IL-2 treatment for immune tolerance induction. For adoptive Treg therapy, “Tregs” normally refers to CD4^+^CD25^+^ T cells, especially in mice. Our data showed that the purity of CD4^+^Foxp3^+^ T cells in CD4^+^CD25^+^ T cells was about 70%, this percentage in published data from GFP reporter mice was even less, about 50% (4). In addition, our data also showed that Tconv preferably expanded during *in vitro* expansion. Hence, it is possible that the big proportion of contaminating CD4^+^Foxp3^-^CD25^+^ effectors might exacerbate the conditions instead of achieving therapeutic purposes. For low-level IL-2 treatment, it is generally believed that CD25, as a unique sub-unit of the IL-2 receptor making Tregs more sensitive to IL-2, is specially expressed on Tregs in the homeostatic state. Using Foxp3 as the definitive Treg marker, the large proportion of CD25^+^Foxp3^-^ Tconv will also be activated and expanded along with Tregs in the presence of low-level of IL-2. This might reduce the efficacy of the inducted Tregs and might even lead to a poorer outcome, especially in strongly inflammatory conditions. Hence, although Treg-based therapies have been shown to be helpful for transplant tolerance induction in animal models, our data suggested that stand-alone adoptive “Treg” therapy or standalone low-level IL-2 therapy might be difficult to move to the clinic.

### 4.4 The expression of CTLA-4 and PD-1 by Tregs and Tconv

Our flow cytometry data showed that the expression of Foxp3 did not completely overlap with the expression of CTLA-4 and PD-1. In other words, although at a higher level in Tregs, both CTLA-4 and PD-1 were constitutively expressed in a small proportion of Tregs and Tconv in the homeostatic state. This suggested that CTLA-4 and PD-1 might work both in Tregs and Tconv to inhibit the activation of the effector arm, although the role of CTLA-4 and PD-1 in Treg-mediated immune-regulation needs to be better defined. CTLA-4 was shown to be crucial for Treg function (12) and has been proposed as a marker to identify regulatory T cells (reviewed in (14)), In contrast, our data showed that only a small proportion of Tregs expressed CTLA-4 even intracellularly and not every Treg expressing CTLA-4 intracellularly also expressed it on the cell surface. Although slightly increased after activation, the expression level and the percentage positive of surface CTLA-4 was maintained at a very low level on Tregs, which suggested that the vast majority of Tregs might not rely on CTLA-4 to mediate immunosuppression.

### 4.5 The higher levels of CTLA-4 and PD-1 in Tregs did not negatively correlate with proliferation compared to Tconv

CTLA-4 and PD-1 serve as co-inhibitory receptors which inhibit T cell activation or increase the activation threshold of T cells. However, the role of CTLA-4 and PD-1 in Treg activation is not fully understood. Although the expression levels of CTLA-4 and PD-1 were higher in/on Tregs than in/on Tconv, there were two unexpected findings: Tregs maintained a higher level of Ki-67 expression in the immune homeostatic state and had greater *in vivo* expansion than Tconv after allo-stimulation in both the adoptive transfer experiment and the skin transplant experiment. This suggested that CTLA-4 and PD-1 did not negatively correlate with Treg activation. Hence, we speculated that either CTLA-4 and PD-1 function differently in Tregs than in Tconv or naïve Tregs were already in an activated state.

### 4.6 Treg specificities

Our data showed that both Tregs and Tconv maintained a basal proliferation as assessed by Ki-67 expression in the homeostatic state. It is possible that this basal proliferation is triggered by self-antigens and identifies T cells that are self-reactive. Although there is some conflicting evidence, the question of whether Tregs are self-reactive or non-self-reactive is still unsettled (reviewed in (14)). Our data showed that one quarter of Tregs and two percent of Tconv were proliferating as measured by their expression of Ki-67. This might be driven by their self-reactivity. In other words, our data supported the argument that most Tregs are non-self-reactive (reviewed in (14)). Contrary to the belief that Tregs are all self-reactive, the non-self-reactivity makes it theoretically feasible for transplant tolerance induction.

### 4.7 The “activated” molecular characteristics of Tregs

CD25 and Ki-67 are commonly used as T cell activation markers and the co-inhibitory molecules, CTLA-4 and PD-1, are upregulated after T cell activation. In this sense, the high expression of these molecules makes Tregs appear like an “activated” Tconv. Our data from differential gene expression further supported this hypothesis. For example, including genes for CD25, CTLA-4, and PD-1 genes for T cell activation markers, inducible co-inhibitory molecules, and key molecules in a number of intracellular activation pathways all were expressed at a higher level in naïve Tregs than naïve Tconv. This pattern of differentially expressed genes between naïve Tregs and naïve Tconv was similar to that between activated Tconv and naïve Tconv. Furthermore, when compared with naïve Tconv, the genes for the suppressor of cytokines which suppress the expression of inflammatory-cytokine-encoding genes were highly expressed in Tregs. In addition to genes encoding CTLA-4, PD-1, and IL-10, the genes encoding their ligands or receptors (e.g., CD86, PD-L1, PD-L2, and IL10R) were also expressed higher in Tregs than in Tconv, which raises the possibility that Tregs might also be target cells of their own mechanisms of action if they were “activated Tconv”. In other words, unless Treg-specific marker(s) are identified from normal animals and humans and Tregs are sufficiently characterised, we cannot preclude another possibility that T cell-mediated immunosuppression is the outcome of self-regulation (reviewed in (14)) in which Tregs might be an over-activated subpopulation which have initiated their negative regulation system such as upregulation of CTLA-4 and PD-1 and release of immunosuppressive cytokines.

In conclusion, our data provides a novel insight into the negative regulation by Tregs and co-inhibitory molecules, the *in vivo* and *in vitro* proliferation characteristics of Tregs compared with Tconv and the molecular characteristics of gene expression in Tregs. However, Treg-specific marker(s) for purification of viable Tregs are badly needed for characterisation and therapeutic studies of Tregs.

## Data availability statement

The datasets presented in this study can be found in online repositories. The names of the repository/repositories and accession number(s) can be found in the article/**Supplementary Material**.

## Ethics statement

The animal study was approved by The University of Newcastle Animal Care and Ethics Committee. The study was conducted in accordance with the local legislation and institutional requirements.

## Author contributions

ZL: Conceptualization, Data curation, Investigation, Methodology, Software, Visualization, Writing – original draft, Formal Analysis. KB: Conceptualization, Data curation, Formal Analysis, Investigation, Methodology, Project administration, Supervision, Validation, Writing – review & editing. NN: Investigation, Methodology, Writing – review & editing. MH: Conceptualization, Funding acquisition, Supervision, Writing – review & editing. DC: Data curation, Methodology, Supervision, Writing – review & editing. GB: Conceptualization, Formal Analysis, Supervision, Writing – review & editing. PT: Conceptualization, Data curation, Formal Analysis, Funding acquisition, Investigation, Methodology, Resources, Supervision, Writing – review & editing.
