# Supplementary figures and images for: Characterizing Foxp3+ and Foxp3- T cells in the homeostatic state and after allo-activation: resting CD4+Foxp3+ Tregs have molecular characteristics of activated T cells
Source: Front Immunol. 2024 Jan 25;15:1292158. doi: 10.3389/fimmu.2024.1292158 (PMC10850883; doi:10.3389/fimmu.2024.1292158)

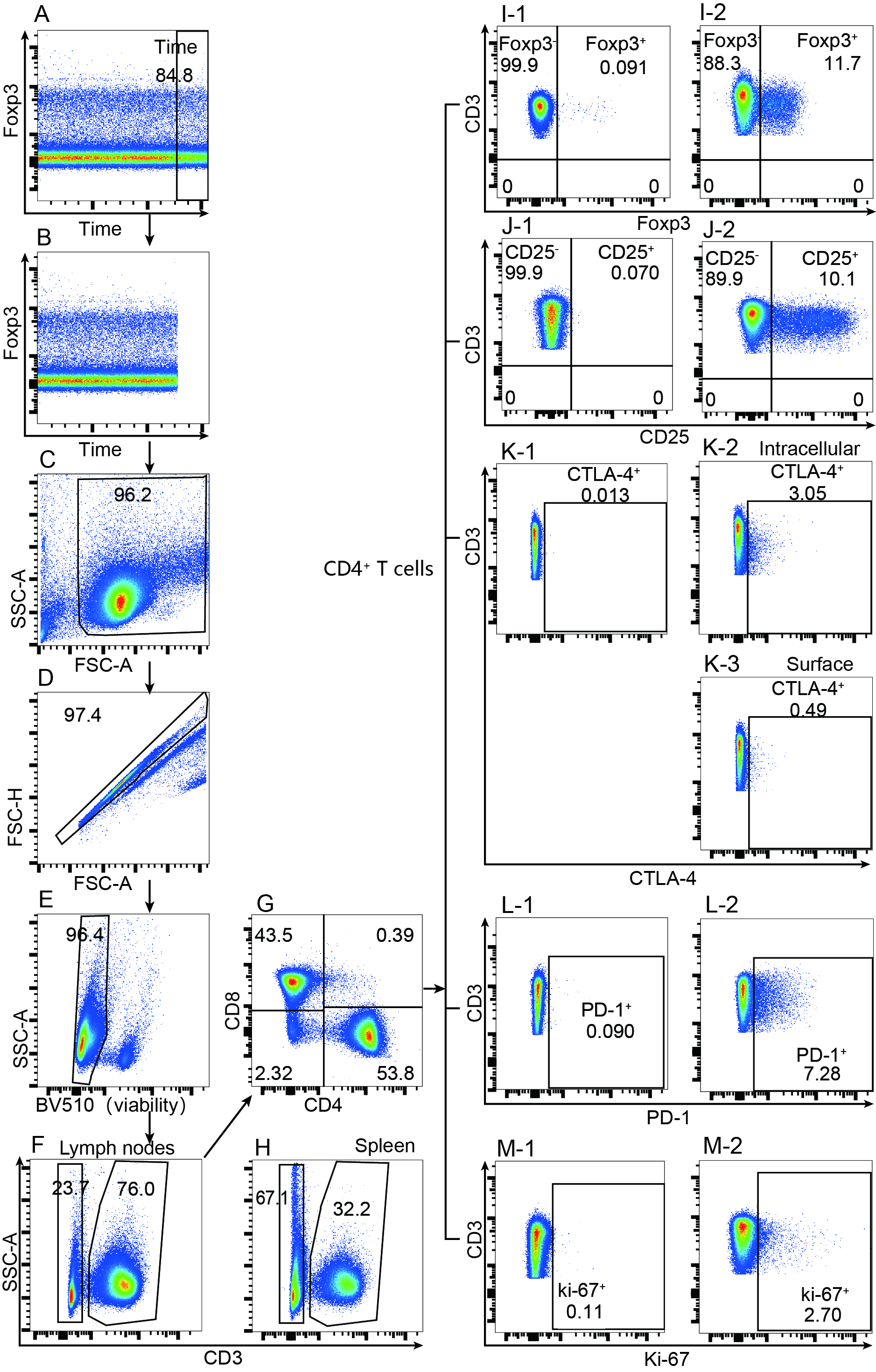

Supplement: Supplementary file 1 [file Image_1.jpeg]

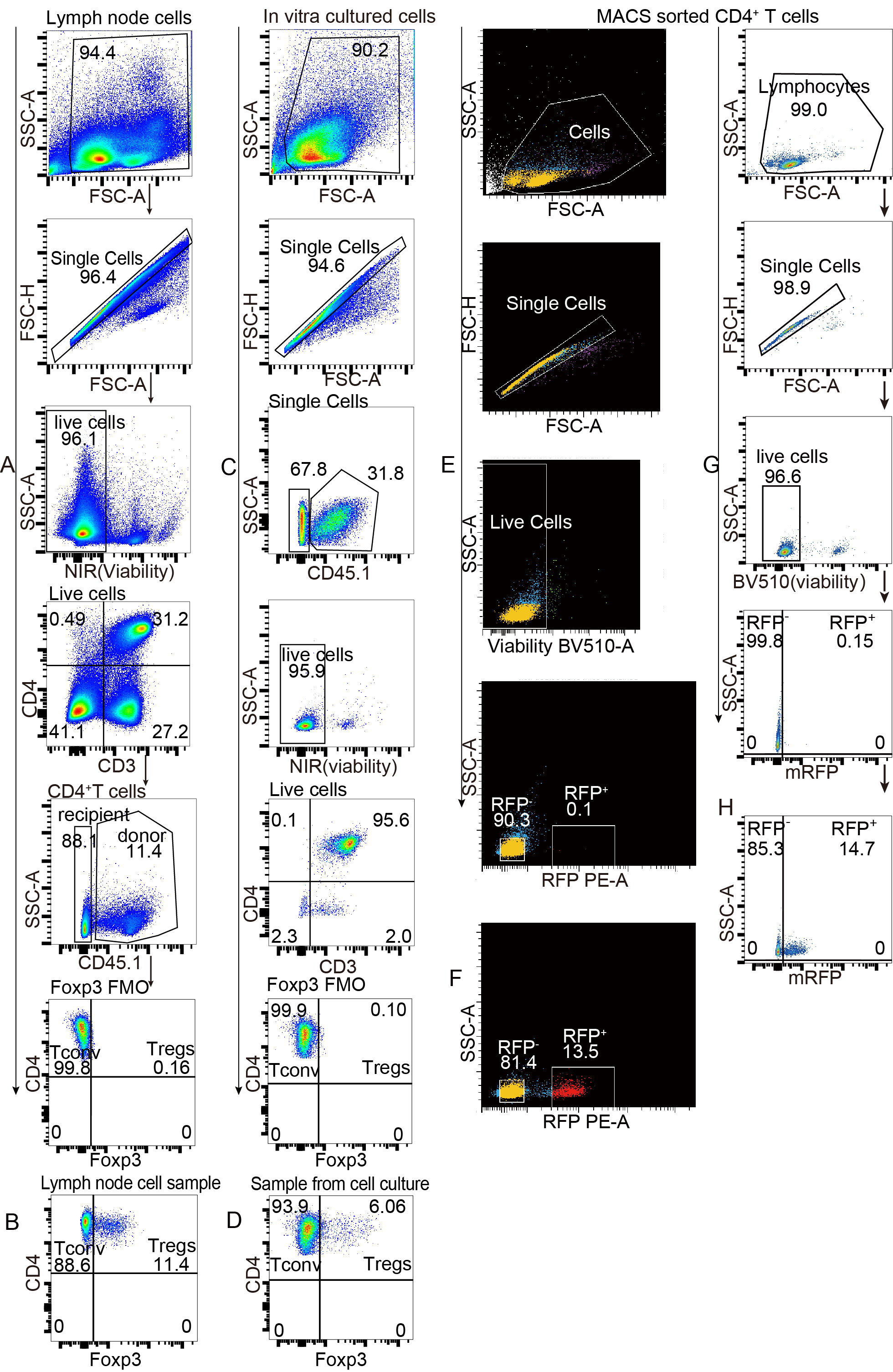

Supplement: Supplementary file 2 [file Image_2.jpeg]
