# Supplementary material for: Characterizing Foxp3+ and Foxp3- T cells in the homeostatic state and after allo-activation: resting CD4+Foxp3+ Tregs have molecular characteristics of activated T cells
Source: Front Immunol. 2024 Jan 25;15:1292158. doi: 10.3389/fimmu.2024.1292158 (PMC10850883; doi:10.3389/fimmu.2024.1292158)
Supplement: Supplementary file 9 [file DataSheet_1.docx]

Supplementary Material

# Supplementary Data

**Supplementary Data 1.** The differentially expressed genes between naive Tregs and naive Tconv by adjusted p < 0.05.

**Supplementary Data 2.** The differentially expressed genes between activated Tconv and naive Tconv by adjusted p < 0.05.

**Supplementary Data 3.** The differentially expressed genes between activated Tregs and naive Tregs by unadjusted p < 0.05.

**Supplementary Data 4.** Raw data for gene expression difference between naive Tregs and naive Tconv.

**Supplementary Data 5.** Raw data for gene expression difference between activated Tconv and naive Tconv.

**Supplementary Data 6.** Raw data for gene expression difference between activated Tregs and naive Tregs.

# Supplementary Figures and Tables

## Supplementary Figures

**Supplementary Figure 1.** Gating strategy for Foxp3, CD25, CTLA-4, PD-1, and Ki-67 in samples from normal and skin graft recipient C57BL/6 mice. (A) and (B) was to remove artifacts due to unstable fluidics if necessary. (C), (D) and (E) were to remove debris, doublets, and dead cells, respectively. (F) and (H) were to show representative T cell percentages in lymph nodes and spleen respectively of normal C57BL/6 mice. (G) was a quadruple gate set on T cells from lymph nodes to generate the four T cell subsets: CD4^+^, CD8^+^, DP and DN T cells. (I-1), (J-1), (K-1), (L-1) and (M-1) were gates set on CD4^+^ T cells for Foxp3, CD25, CTLA-4, PD-1 and Ki-67 respectively using fluorescence minus one (FMO) samples. The gates in (I-2), (J-2), (L-2) and (M-2) were copied from (I-1), (J-1), (L-1) and (M-1) respectively an experimental sample from lymph nodes. The gates in (K-2) and (K-3) were copied from (K-1) in samples stained with CTLA-4 intracellularly and on cell surface respectively. Samples from lymph nodes are used for this demonstration except H.

**Supplementary Figure 2.** Gating strategies for Tregs and Tconv in in vivo and in vitro expansion, cell sorting and re-analysis of the cell sorting data in Flowjo 10.9. Any unstable fluidics were removed as shown in Supplementary Figure 1. (A) shows the sequential gating for donor CD4^+^Foxp3^+^ Tregs and CD4^+^Foxp3^-^ Tconv in adoptive transfer or in vivo expansion experiment. The gate for Tregs and Tconv in (B) using a lymph node sample was copied from Foxp3 FMO in (A). Likewise, (C) shows the sequential gating for responder CD4^+^Foxp3^+^ Tregs and CD4^+^Foxp3^-^ Tconv in cell culture or in vitro expansion experiment. The gate for Tregs and Tconv in (D) using an experiment sample was copied from Foxp3 FMO in (C). (E) and (F) show the cell sorting layout in FACSDiva. CD4^+^Foxp3^+^mRFP^+^ Tregs and CD4^+^Foxp3^-^mRFP^-^ Tconv were sorted from pre-enriched CD4^+^ T cells from mRFP reporter mice. (E) shows a conservative gate for Tregs (mRFP^+^) and Tconv (mRFP^-^) in a mRFP FMO sample (CD4^+^ T cells from normal C57BL/6 mice stained with viability dye). These samples were used in the differential gene expression experiment. The gate in (F) using an experiment sample is the same gate in (E). To compare the expression of mRFP and Foxp3, the data in (E) and (F) were re-analyzed in Flowjo 10.9 as shown in (G) and (H). (G) is the sequential gating for Tregs and Tconv using a FMO sample. The gate in (H) was copied from FMO sample in (G).
